# Effect of Endometrial Thickness Change in Response to Progesterone Administration on Pregnancy Outcomes in Frozen-Thawed Embryo Transfer: Analysis of 4465 Cycles

**DOI:** 10.3389/fendo.2020.546232

**Published:** 2020-10-29

**Authors:** Jing Ye, Jie Zhang, Hongyuan Gao, Yanwen Zhu, Yao Wang, Renfei Cai, Yanping Kuang

**Affiliations:** Department of Assisted Reproduction, Shanghai Ninth People’s Hospital, Shanghai Jiaotong University School of Medicine, Shanghai, China

**Keywords:** endometrial thickness change, progesterone, clinical pregnancy rate (CPR), live birthrates, transvaginal ultrasound

## Abstract

**Objective:**

To evaluate whether endometrial thickness (EMT) change in response to progesterone has an effect on pregnancy outcomes in frozen-thawed embryo transfer (FET) cycles.

**Design:**

Retrospective observational study.

**Setting:**

Tertiary-care academic medical center.

**Participants:**

4465 infertile women undergoing their first FET between January 2010 and December 2015 in our center.

**Methods:**

This observational study included 4465 patients undergoing their first FET cycles between January 2010 and December 2015. EMT was measured by transvaginal ultrasound one day before progesterone administration and on the day of FET to observe EMT change.

**Main outcome measures:**

Clinical pregnancy rate (CPR) and the live birthrate (LBR) was discussed.

**Results:**

Regardless of the endometrial preparation protocols such as artificial cycle, estrogen-progesterone replacement therapy (EP) or natural cycle (NC), EMT may increase, decrease or remain stable on the day of FET compared with that of one day before progesterone administration. CPR in EMT increase, decrease and stable groups were 48.4%, 51.3% and 50.7% in EP cycle versus 49.2%, 52.0% and 48.9% in NC cycle, showing no significant difference between the three groups in both cycles (P= 0.48, P= 0.49). LBR was 40.9%, 45.9% and 42.6% in EP cycle versus 44.2%, 44.8% and 42.1% in NC cycle, also showing no significant difference between the three groups in both cycles (P= 0.16, P= 0.66). In addition, CPR and LBR were not significantly associated with EMT increase.

**Concludes:**

EMT may increase, decrease or remain stable on the day of FET as compared with that of one day before progesterone administration. Whatever change in EMT that occurs after progesterone administration has no significant effect on CPR and LBR in FET cycles.

## Introduction

The breakthrough in assisted reproductive technology (ART) has allowed couples previously unable to conceive to get pregnant. For a pregnancy to be made, the embryo must implant in a receptive endometrium during the window of implantation, which is around 7 days after ovulation of a menstrual cycle (1). In vitro fertilization (IVF) has allowed security of the embryonic development. Therefore, the focus of the debate has been placed on endometrial receptivity and IVF outcomes. Sonography is a noninvasive method which has been used to evaluate endometrial receptivity. Several sonographic parameters include endometrial thickness (EMT) (2, 3), endometrial pattern (4, 5), and endometrial blood flow (6–8), have been evaluated during the embryo transfer cycle.

EMT has been broadly acknowledged as a prognostic indicator for endometrial receptivity, but the results are controversial (2, 9–13). In most previous studies, EMT was monitored during ovarian stimulation in fresh embryo transfer cycles, or during the endometrium proliferation phase in frozen-thawed embryo transfer (FET) cycles, or in the luteal phase on the embryo transfer day (2, 9–13).

The physiology of the endometrium is changeable and serves as the distinction between the follicular and luteal phases (14–16). A typical change of the endometrial pattern is the change from pattern A (a triple-line pattern), pattern B (an intermediate isoechogenic pattern), to pattern C (homogenous, hyperechogenic endometrium) (4). Many published studies have evaluated the correlation between the endometrial pattern and pregnancy outcomes, with conflicting conclusions (4, 7, 16–18). Few studies have been aware of the relationship between EMT change after progesterone administration and the pregnancy outcome.

This study aimed to observe EMT change in response to progesterone and to explore the impact of EMT change after progesterone administration on the pregnancy outcome in FET cycles.

## Materials and Methods

### Study Design and Patients

This retrospective study was performed at the Department of Assisted Reproduction of the Ninth People’s Hospital of Shanghai Jiao Tong University School of Medicine (Shanghai, China), involving 4465 women who underwent their first FET cycle during the period between January 2010 and December 2015 in our center. The inclusion criteria were women aged ≤40 years with a body mass index (BMI)<30 kg/m^2^ who underwent their first FET cycle in our center and embryo transfer on day 3. The exclusion criteria were women with uterine malformation, endometriosis, adenomyosis, and endometrial polyps or submucosae myomas as determined hysteroscopy that may affect embryo implantation. The study was observational only, with no intervention, and was approved by the Ethics Committee (Institutional Review Board) of Shanghai Ninth People’s Hospital.

### Treatment Protocol

Endometrial preparation for FET included an artificial cycle (estrogen-progesterone cycle, EP) and natural cycles (NCs). For NCs, all patients underwent ultrasonic evaluation from day 10 of the menstrual cycle. The EMT and mean diameter of the dominant follicle were examined by the same doctor. When the diameter of the dominant follicle was about 18mm and EMT reached 7mm, a blood sample was obtained for progesterone, estradiol (E_2_) and luteinizing hormone (LH) level detection. When E_2_ was >150 pg/mL and progesterone was <1 ng/mL, one of two procedures was performed depending on the serum LH value: for LH <20 mIU/mL, a bolus of 5,000 IU human chorionic gonadotropin (hCG) was administered on the same night, progesterone was given 3 days later, and the cleavage-stage embryo was transferred 5 days later after hCG injection. If the serum LH concentration was ≧20 mIU/mL, hCG was administered on the same afternoon, and progesterone was given 2 days later. The cleavage-stage embryo was transferred 4 days later. The EMT on the day of hCG administration was recorded by transvaginal ultrasound.

For EP cycles, the patients began sequential oral administration of micronized estradiol (8mg/day) on cycle day 3. EMT ultrasound measurement was performed 12-14 days after the initiation of micronized estradiol. Progesterone was administered when EMT reached 7mm. The patients commenced oral administration of yellow Fematon tablets, 2# bid, including 2 mg micronized estradiol and 10 mg dydrogesterone per tablet (Abbott Healthcare Products B.V.) and soft vaginal P capsules, 200 mg bid (Laboratoires Besins International). EMT on the day before progesterone administration was also recorded by transvaginal ultrasound. Embryos were warmed and transferred on day 3 of P supplementation. A maximum of two embryos were allowed for transfer.

Details about the endometrial preparation for FET and EMT measurement method can be found in previous studies (19, 20).

### EMT Assessment

Based on differences in EMT measurement between the day of embryo transfer and the day before progesterone administration, the patients were divided into three groups: patients with increased EMT (EMT increase group), patients with decreased EMT (EMT decrease group), and patients with stable EMT (No EMT change group).

All ultrasound measurements were performed by a senior technician using GE Voluson E8 (GE Healthcare, Austria). EMT was defined by the maximal distance from one endometrial-myometrial interface to the other in the mid-sagittal plane in accordance with our previous work (19, 20). In NCs, EMT before progesterone administration was recorded from the day of hCG administration, while in EP cycles, it was recorded from the final ultrasound before initiation of progesterone administration. For all patients, the endometrium was also re-evaluated on the morning of the embryo transfer day to exclude endometrial cavity fluid and other unfavorable conditions that were appropriate for embryo transfer.

### Laboratory Protocols

All embryos were cultured under mineral oil in incubator at 37°C, under 5% O2 and 6% CO2 concentration. The most widely used criteria for selecting the best embryos for transfer have been based on cell number and morphology (21). All cleavage embryos were evaluated by an experienced embryologist on Day 3 with the use of the grading system proposed by Cummins, J. M in 1986 (21) in our center. Embryo grading in our center (grading I~IV, represents a decline in embryo quality): Grade I: embryos with the regularity or symmetry of blastomere size, uniform and clear without particles in the cytoplasm, and ≤ 10% fragmentation in the perivitelline space. Grade II: embryos with the regularity or symmetry of blastomere size, the presence or absence of particles in the cytoplasm, and fragmentation in the perivitelline space range from 10-20%. Grade III: embryos with the apparent irregularity or dissymmetry of blastomere size, the apparent particles in the cytoplasm, and fragmentation in the perivitelline space range from 20-50%. Grade IV: embryos with the severe irregularity or dissymmetry of blastomere size, the severe particles in the cytoplasm, and >50% fragmentation in the perivitelline space. Only Grade I and Grade II embryos were selected for cryopreservation on Day 3. Grade III and IV embryos were continued to culture until blastocyst stage.

The method of embryo cryopreservation was all vitrification in our center. Embryo vitrification was performed *via* Cryotop carrier system, in conjunction with dimethylsulfoxide–ethylene glycol–sucrose as cryoprotectants. For thawing, embryos were transferred into dilution solution in a sequential manner (1 mol/L to 0.5 mol/L to 0 mol/L sucrose).

### Statistical Analysis

Serum hCG was detected 14 days after embryo transfer to assess the FET outcome. Once pregnancy was achieved, the exogenous progesterone supplement as luteal support was continued until 10 weeks of gestation. Clinical pregnancy was confirmed by ultrasound observation as the presence of at least one gestational sac in the uterine cavity at 4 weeks after FET. The live birthrate (LBR) was considered as those cycles resulting in the delivery of infants after 24 weeks of gestation and expressed per FET cycle. Spontaneous abortion was defined as spontaneous pregnancy loss after sonography visualization of an intrauterine gestational sac and is expressed per clinical pregnancy cycle. The diagnosis of ectopic pregnancy (EP) is at least one gestational sac outside the uterine cavity by ultrasound observation. Heterotopic pregnancy, which is described as a co-existence of an intra- and extra-uterine gestation sac, was also classified as an EP in our study. The incidence of EP was based on the number of EP cycles per 100 clinical pregnancy tests after FET.

In the study, data are presented in the mean± standard deviation (SD). Data were analyzed using one-way analysis of variance for continuous data, and χ2-test or Fisher’s exact tests for categorical data, as appropriate. The Mann-Whitney U-test was used for the non-normal distribution. P<0.05 was considered statistically significant. All data were analyzed using the Statistical Package for the Social Sciences for Windows (SPSS, ver. 25.0).

## Results

In this observational study, 4465 first FET cycles were recorded. The baseline demographic and cycle characteristics of all patients are shown in **Table 1** according to EMT changes. In all FET cycles, EMT was decreased in 27.6% patients and increased in 46.1% patients on the day of embryo transfer.

**Table 1:** Basic characteristics and pregnancy outcomes in women transferred with Day 3 frozen-thawed embryos.

	Decreasing	No change	Increasing	P
No. of cycles, n (%)	1231(27.6)	1172(26.3)	2062(46.1)	
Age(year)	29.97 ± 2.71	29.54 ± 2.46	29.83 ± 2.71	0.25^a^
Duration of infertility(year)	3.40 ± 2.16	3.41 ± 2.22	3.44 ± 2.15	0.49^a^
Previous IVF attempts, n, (%)				0.42^b^
0	1029(83.6)	991(84.6)	1753(85.0)	
1-2	123(10.0)	109(9.3)	206(10.0)	
≥3	79(6.4)	72(6.1)	103(5.0)	
BMI (Kg/m^2^)	21.37 ± 2.67	21.18 ± 2.62	21.24 ± 2.65	0.24^a^
Infertility diagnosis n, (%)				0.21^b^
Female factor	695(56.5)	652(55.6)	1133(54.9)	
Male factor	159(12.9)	173(14.8)	316(15.3)	
Unexplained	80(6.5)	76(6.5)	163(7.9)	
Combined factors	297(24.1)	271(23.1)	450(21.8)	
Endometrial thickness: starting progesterone	11.03 ± 2.07	11.09 ± 2.25	10.06 ± 1.69	0.00^a^
Endometrial thickness: embryo transfer	9.75 ± 1.84	11.09 ± 2.25	11.91 ± 2.21	0.00^a^
No. of embryos transferred n, (%)				0.41^b^
1	83(6.7)	90(7.7)	165(8.0)	
2	1148(93.3)	1082(92.3)	1897(92.0)	
No. of transferred embryos grading, n (%)				0.14^b^
Grade I	336(14.1)	333(14.8)	630(15.9)	
Grade II	2043(85.9)	1921(85.2)	3329(84.1)	
Year of treatment				0.14^b^
2010-2011	137(11.1)	152(13.0)	261(12.7)	
2012-2013	242(19.7)	265(22.6)	448(21.7)	
2014-2015	852(69.2)	755(64.4)	1353(65.6)	
Clinical pregnancy rate n, (%)	615(50.0)	605(51.6)	1007(48.8)	0.31^b^
Abortion rate n, (%)	87(14.1)	70(11.6)	120(11.9)	0.33^b^
Ectopic pregnancy rate n, (%)	6(1.0)	4(0.7)	9(0.9)	0.86^c^
Live birth rate n, (%)	522(42.4)	531(45.3)	878(42.6)	0.25^b^

a One-way ANOVA. Values are mean ± SD.

b Pearson chi-square test. Values are the number (percentage).

c Fisher’s exact test. Values are the number (percentage).

**Table 2** shows the basic demographic characteristics and pregnancy outcomes in FET cycles according to EMT changes and the endometrial preparation protocol. Regardless of the endometrial preparation protocol, maternal age and BMI, etiology of infertility treatment, the infertility duration, previous IVF tries and the number of embryos transferred were comparable in the three groups. However, EMT on the day before progesterone administration in EMT increase group was the thinnest of the three groups (9.97 ± 1.60 *vs.* 10.55 ± 2.14 *vs.* 10.74 ± 1.98mm, P= 0.00 in EP cycles; 10.15 ± 1.78 *vs.* 11.58 ± 2.23 *vs.* 11.47 ± 2.12mm, P= 0.00 in NC cycle); it increased dramatically and became the thickest on the day of embryo transfer when compared to the other two groups (11.58 ± 2.06 *vs.* 10.55 ± 2.14 *vs.* 9.51 ± 1.80, P= 0.00 in EP cycles; 12.21 ± 2.30 *vs.* 11.58 ± 2.23 *vs.* 10.11 ± 1.84, P= 0.00 in NC cycles).

**Table 2:** Basic characteristics and pregnancy outcomes in women treated with different endometrial preparation protocols during FET cycles.

	E-P cycle (N=2293)				Natural cycle (N=2172)			
	Decreasing	No change	Increasing	P	Decreasing	No change	Increasing	P
No. of cycles, n (%)	742(32.4)	560(24.4)	991(43.2)		489(22.5)	612(28.2)	1071(49.3)	
Age(year)	29.95 ± 2.62	29.65 ± 2.42	29.95 ± 2.73	0.07^a^	29.99 ± 2.85	29.44 ± 2.49	29.71 ± 2.69	0.08^a^
Duration of infertility(year)	3.41 ± 2.15	3.45 ± 2.21	3.51 ± 2.15	0.45^a^	3.40 ± 2.18	3.38 ± 2.23	3.39 ± 2.15	0.80^a^
History of prior gravidity (%)	394(53.1)	283(50.5)	548(55.3)	0.19^b^	177(36.2)	244(39.9)	369(34.5)	0.08^b^
Previous IVF attempts, n (%)				0.30^b^				0.99^b^
0	601(81.0)	450(80.4)	809(81.6)		428(87.5)	541(88.4)	944(88.1)	
1-2	81(10.9)	62(11.1)	122(12.3)		42(8.6)	47(7.7)	84(7.8)	
≥3	60(8.1)	48(8.6)	60(6.1)		19(3.9)	24(3.9)	43(4.0)	
BMI (Kg/m^2^)	21.60 ± 2.71	21.48 ± 2.76	21.48 ± 2.79	0.47^a^	21.03 ± 2.57	20.91 ± 2.46	21.03 ± 2.49	0.77^a^
Infertility diagnosis, n (%)				0.10^b^				0.90^b^
Female factor	436(58.8)	325(58.0)	573(57.8)		259(53.0)	327(53.4)	560(52.3)	
Male factor	83(11.2)	72(12.9)	142(14.3)		76(15.5)	101(16.5)	174(16.2)	
Unexplained	43(5.8)	40(7.1)	81(8.2)		37(7.6)	36(5.9)	82(7.7)	
Combined factors	180(24.3)	123(22.0)	195(19.7)		117(23.9)	148(24.2)	255(23.8)	
Endometrial thickness: starting progesterone	10.74 ± 1.98	10.55 ± 2.14	9.97 ± 1.60	0.00^a^	11.47 ± 2.12	11.58 ± 2.23	10.15 ± 1.78	0.00^a^
Endometrial thickness: embryo transfer	9.51 ± 1.80	10.55 ± 2.14	11.58 ± 2.06	0.00^a^	10.11 ± 1.84	11.58 ± 2.23	12.21 ± 2.30	0.00^a^
No. of embryos transferred, n (%)				0.82^b^				0.42^b^
1	49(6.6)	42(7.5)	70(7.1)		34(7.0)	48(7.8)	95(8.9)	
2	693(93.4)	518(92.5)	921(92.9)		455(93.0)	564(92.2)	976(91.1)	
No. of transferred embryos grading, n (%)				0.09^b^				0.80^b^
Grade I	202(14.1)	161(14.9)	321(16.8)		134(14.2)	172(14.6)	309(15.1)	
Grade II	1233(85.9)	917(85.1)	1591(83.2)		810(85.8)	1004(85.4)	1738(84.9)	
Year of treatment				0.29^b^				0.21^b^
2010–2011	75(10.1)	73(13.0)	132(13.3)		62(12.7)	79(12.9)	129(12.0)	
2012–2013	148(19.9)	112(20.0)	202(20.4)		94(19.2)	153(25.0)	246(23.0)	
2014–2015	519(69.9)	375(67.0)	657(66.3)		333(68.1)	380(62.1)	696(65.0)	
Clinical pregnancy rate, n (%)	376(50.7)	287(51.3)	480(48.4)	0.49^b^	239(48.9)	318(52.0)	527(49.2)	0.48^b^
Abortion rate n, (%)	57(15.2)	29(10.1)	70(14.6)	0.13^b^	30(12.6)	42(13.2)	52(9.9)	0.28^b^
Ectopic pregnancy rate, n (%)	3(0.8)	1(0.3)	5(1.0)	0.53^c^	3(1.3)	2(0.6)	2(0.4)	0.37^c^
Live birth rate, n (%)	316(42.6)	257(45.9)	405(40.9)	0.16^b^	206(42.1)	274(44.8)	473(44.2)	0.66^b^

a One-way ANOVA. Values are mean ± SD.

b Pearson chi-square test. Values are the number (percentage).

c Fisher’s exact test. Values are the number (percentage).

**Figure 1:**
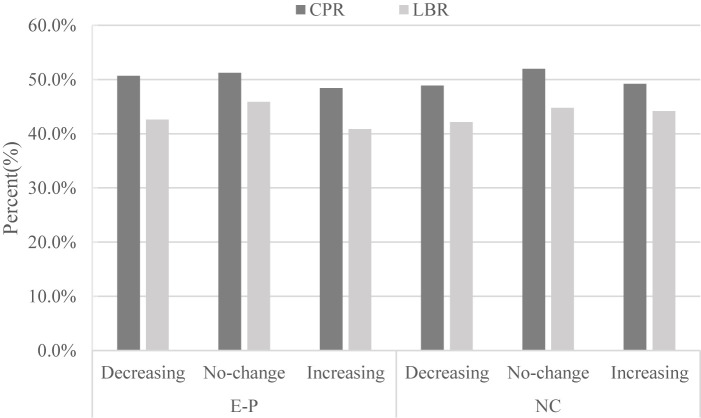
Relationship between endometrium change in response to progesterone and pregnancy outcomes (clinical pregnancy rate and live birth rate). E-P, estrogen-progesterone cycle; NC. natural cycle.

As shown **Table 2** and **Figure 1**, there was no significant difference in the pregnancy outcome in terms of the clinical pregnancy rate (CPR) (48.4% *vs.* 51.3% *vs.*50.7%, P= 0.49 in EP cycles; 49.2% *vs.* 52.0% *vs.* 48.9%, P= 0.48 in NC cycles) and live birthrates (40.9% *vs.* 45.9% *vs.* 42.6%, P= 0.16 in EP cycles; 44.2% *vs.* 44.8% *vs.*42.1%, P= 0.66 in NC cycles) whether EMT increased, decreased or remained stable. Besides, the abortion and EP rates were also comparable between the three groups irrespective of EP or NC cycles.

**Figure 2:**
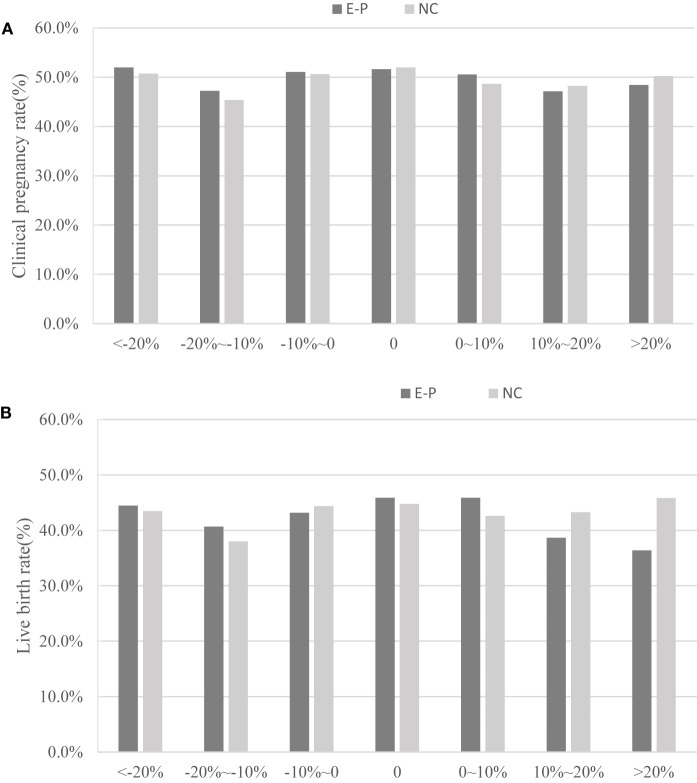
Relationship between the endometrium change ratio and pregnancy outcomes (clinical pregnancy rate and live birth rate). E-P, estrogen-progesterone cycle; NC, natural cycle. **(A)** represents clinical pregnancy rate; **(B)** represents the live birth rate.

**Table 3** briefly depicts the EMT on the day before progesterone administration and the day of embryo transfer in patients with different EMT change ratios. Regardless of EP or NC cycles, EMT in endometrial thickness increasing ≥20% group before progesterone administration was the thinnest of the three groups. It also increased dramatically and became the thickest on the day of embryo transfer when compared to all other groups. **Table 3** and **Figure 2** clearly show that CPR (P= 0.46 in EP cycles; P= 0.81 in NC cycles) and LBR (P= 0.07 in EP cycles; P= 0.75 in NC cycles) did not change significantly with the increase ratio of EMT change.

**Table 3:** Endometrial thickness on the day before progesterone administration/embryo transfer and pregnancy outcomes according to the endometrial thickness change ratio.

	Endometrial change ratio	<-20%	-20%~-10%	-10%~0	0	0~10%	10%~20%	>20%
EP cycle	No. of cycles(n)	108	231	403	560	401	274	316
	Age (year)	29.85 ± 2.42	30.03 ± 2.68	29.94 ± 2.65	29.65 ± 2.42	29.93 ± 2.69	30.19 ± 2.73	29.77 ± 2.78
	EMT on dPA (mm)	11.44 ± 2.12	11.07 ± 1.97	10.36 ± 1.86	10.55 ± 2.14	10.19 ± 1.62	9.91 ± 1.61	9.75 ± 1.53
	EMT on dET (mm)	8.38 ± 1.64	9.47 ± 1.67	9.83 ± 1.79	10.55 ± 2.14	10.74 ± 1.71	11.40 ± 1.84	12.82 ± 2.05
	Clinical pregnancy rate	52.0%	47.2%	51.1%	51.6%	50.5%	47.1%	48.4%
	Live birth rate	44.4%	40.7%	43.2%	45.9%	45.9%	38.7%	36.4%
NC cycle	No. of cycles(n)	69	163	257	612	331	282	458
	Age (year)	29.78 ± 2.83	30.31 ± 2.89	29.84 ± 2.82	29.44 ± 2.49	29.59 ± 2.80	29.85 ± 2.64	29.72 ± 2.65
	EMT on dPA (mm)	12.94 ± 2.61	11.50 ± 2.08	11.05 ± 1.80	11.58 ± 2.23	10.58 ± 1.91	10.25 ± 1.68	9.78 ± 1.65
	EMT on dET (mm)	9.57 ± 2.14	9.79 ± 1.75	10.46 ± 1.73	11.58 ± 2.23	11.16 ± 2.02	11.80 ± 1.96	13.23 ± 2.26
	Clinical pregnancy rate	50.7%	45.4%	50.6%	52.0%	48.6%	48.2%	50.2%
	Live birth rate	43.5%	38.0%	44.4%	44.8%	42.6%	43.3%	45.9%

EP, estrogen-progesterone; NC, natural cycle; EMT, endometrial thickness; dPA, the day before progesterone administration; dET, day of embryo transfer; Endometrial thickness change ratio = (Thickness on dET-Thickness on dPA)/Thickness on dPA*100%.

## Discussion

In this retrospective study, we demonstrated that EMT change in response to progesterone administration had no significant impact on the pregnancy outcome in FET cycles. Additionally, the data also showed no significant difference in CPR and LBR with the increasing ratio of EMT change regardless of the endometrial preparation protocol (p>0.05).

Although many studies have addressed the impact of EMT on the pregnancy outcome, data for the measurement of endometrial characteristics during each IVF cycle varied between these studies. One of the reasons for these discrepancies is that different investigators measured EMT in different time points, either on the day of hCG administration (3, 22, 23), or on the day of oocyte retrieval (24), or on the day of embryo transfer (13, 25), which makes it difficult to compare between studies.

In most aforementioned studies, EMT was measured during the proliferation phase before progesterone administration. However, the physiology of endometrial development is distinctive between the follicular and luteal phases. In the follicular phase, the endometrium is susceptible to estrogen, which increases EMT and accelerates the linear growth of endometrial glands and blood vessels (14). The endometrial proliferation ceases 2-3 days after ovulation under the influence of P (14). Few studies have been aware of the relationship between EMT change after progesterone administration and the pregnancy outcome.

Only a few studies have evaluated EMT change during IVF stimulation (4, 15, 16, 26–28), but some of their conclusions are conflicting. Zhao et al. reported that EMT change between the third day of gonadotrophin stimulation and the day of hCG administration was not prognostically useful in predicting the occurrence of pregnancy, and combination detection of the EMT change and type could not predict the clinical outcome correctly, either (4). But regrettably, they failed to investigate the relationship between EMT change after progesterone administration and the pregnancy outcome. Haas, J. et al. examined the difference in EMT between the late estrogen phase and the day of embryo transfer in 274 FET cycles and found that there was a highly significant inverse correlation between the pregnancy outcome and EMT change (26). However, there were a few weaknesses in their study. First, they calculated the EMT by using different measurement methods: they used transabdominal ultrasound on the day embryo transfer day, and vaginal ultrasound at the end of the estrogen phase. It is common knowledge that transvaginal ultrasound is more accurate than transabdominal ultrasound for the measurement of EMT. Second, they only included women undergoing the hormonal preparation protocol for the FET cycle, and therefore the results may not be generalized to women in ovulatory FET cycles. Third, they only evaluated the ongoing pregnancy rate in the FET cycle and did not describe the relationship between EMT change in response to progesterone administration and LBR. All these defects may be the main reasons for the inconsistency with our results. Bu et al. (27) found that EMT kept increasing or remained stable after progesterone administration. They also reported that an increased endometrium after progesterone administration was associated with a better pregnancy outcome. Haas et al. (26) even reported that the endometrium may compact (endometrial thickness becomes thinner) after progesterone administration. Bu’s study did not describe the relationship between endometrial compaction and the pregnancy outcome, neither did they depict the relationship between EMT change in response to progesterone administration and LBR.

The present study attempted to overcome the flaws of those previous studies and examine the impact of EMT change in response to progesterone administration on the pregnancy outcome in FET cycles. Based on 4465 FET cycles, our results demonstrated that there was no significant difference in CPR and LBR regardless of EMT increasing, decreasing or remaining stable after progesterone administration. In addition, the abortion rate and EP rate were also comparable between the three groups irrespective of EP or NC cycles. Likewise, CPR and LBR did not undergo significant changes with the increasing ratio of EMT regardless of the endometrial preparation protocol in FET cycles.

There were several limitations in this study. Our study was restricted by its retrospective design, although we fastidiously reviewed our database with strict inclusion and exclusion criteria. Also, we further restricted the analysis to the pregnancy outcomes on day 3 embryo transfers to eliminate possible impact of the culture duration on the FET outcome (29). The main strength of this study was that the data for the FET cycles came from a single center, where practice consistency can be assured. Throughout the study period, aside from laboratory conditions, endometrial preparation protocols were also very consistent. Additionally, the endometrial thickness of each patient was measured by the same doctor in our center during the study period. The variability of inter-observer and the same observer in different time periods in the measurement of the endometrial thickness may have brought some bias to our study.

## Conclusion

The present single-center large-sample study may expand the current knowledge about the effect of EMT change in response to progesterone administration on the pregnancy outcome in FET cycles. In both EP and NC cycles for endometrium preparation protocols, EMT on the day of embryo transfer may increase, decrease, or remain stable. The results of the present study demonstrated that there was no significant difference in CPR and LBR regardless of EMT increasing, decreasing or remaining stable after progesterone administration. Additionally, CPR and LBR did not undergo significant changes with the increasing ratio of EMT change regardless of EP or NC cycles. Nevertheless, further better-designed and powered randomized clinical trials are needed to confirm these retrospective findings.

## Data Availability Statement 

The raw data supporting the conclusions of this article will be made available by the authors, without undue reservation.

## Ethics Statement

The studies involving human participants were reviewed and approved by the institutional review board of the Ninth People’s Hospital of Shanghai Jiao Tong University School of Medicine. The patients/participants provided their written informed consent to participate in this study.

## Author Contributions

YK and RC supervised the entire study, including procedures, conception, design, and completion. HG, YZ, and YW were responsible for collecting information. JY and JZ contributed to the analysis data and drafted the manuscript. JY and JZ contributed equally to this article. All authors took part in the ultimate interpretation of the study data and manuscript revisions. All authors contributed to the article and approved the submitted version.

## Funding

The National Key Research and Development Program of China (2018YFC1003000); the National Natural Science Foundation of China (81771533).

## Conflict of Interest

The authors declare that the research was conducted in the absence of any commercial or financial relationships that could be construed as a potential conflict of interest.
